# The cortical structure of functional networks associated with age-related cognitive abilities in older adults

**DOI:** 10.1371/journal.pone.0204280

**Published:** 2018-09-21

**Authors:** Michael B. Kranz, Michelle W. Voss, Gillian E. Cooke, Sarah E. Banducci, Agnieszka Z. Burzynska, Arthur F. Kramer

**Affiliations:** 1 Department of Psychology, University of Illinois at Urbana Champaign, Urbana, IL, United States of America; 2 Beckman Institute for Advanced Science and Technology, University of Illinois at Urbana Champaign, Urbana, IL, United States of America; 3 Department of Psychological and Brain Sciences, University of Iowa, Iowa City, IA, United States of America; 4 Department of Human Development and Family Studies/ Molecular, Cellular and Integrative Neurosciences, Colorado State University, Fort Collins, CO, United States of America; 5 Departments of Psychology and Mechanical and Industrial Engineering, Northeastern University, Boston, MA, United States of America; Nathan S Kline Institute, UNITED STATES

## Abstract

Age and cortical structure are both associated with cognition, but characterizing this relationship remains a challenge. A popular approach is to use functional network organization of the cortex as an organizing principle for *post-hoc* interpretations of structural results. In the current study, we introduce two complimentary approaches to structural analyses that are guided by *a-priori* functional network maps. Specifically, we systematically investigated the relationship of cortical structure (thickness and surface area) of distinct functional networks to two cognitive domains sensitive to age-related decline thought to rely on both common and distinct processes (executive function and episodic memory) in older adults. We quantified the cortical structure of individual functional network’s predictive ability and spatial extent (i.e., number of significant regions) with cognition and its mediating role in the age-cognition relationship. We found that cortical thickness, rather than surface area, predicted cognition across the majority of functional networks. The default mode and somatomotor network emerged as particularly important as they appeared to be the only two networks to mediate the age-cognition relationship for both cognitive domains. In contrast, thickness of the salience network predicted executive function and mediated the age-cognition relationship for executive function. These relationships remained significant even after accounting for global cortical thickness. Quantifying the number of regions related to cognition and mediating the age-cognition relationship yielded similar patterns of results. This study provides a potential approach to organize and describe the apparent widespread regional cortical structural relationships with cognition and age in older adults.

## Introduction

Cognitive neuroscience of aging links well-established effects of aging on cognition to the effects of aging on brain structure and function [1]. The functional interactions of regions in cognitively-relevant networks, such as the default mode (DMN), control, dorsal attention, and salience networks, decline during aging [2–8]. Therefore, functional network-based approaches have emerged as a powerful organizing principle to describe the role of brain function in cognitive aging.

In contrast, the relationship between structure and cognition in older adults remains less understood. Several studies have found associations between regional cortical volume, cognition, and age within anatomically-defined regions of interest, particularly within the prefrontal cortex [9–11]. However, these prefrontal volumetric measures have shown a limited relationship with age-related cognition over and above global volumetric measures [12] and have negatively predicted cognition in some older adult samples, leaving uncertainty as to what the metric of cortical volume indexes [13,14]. Moreover, it appears age-related decreases in brain volume extend beyond more traditional cortical parcellations [15,16]. Taken together, alternative organizing principles (rather than maps based on anatomical landmarks) and measures of cortical structure (rather than volume) must be investigated, especially in older adults.

One potential issue with this approach is that cortical volume is the product of cortical surface area and thickness, which measure distinct attributes of the brain [17–19]. Surface area expansion during development is thought to bring interconnected regions closer together—a process thought to increase brain connectivity more efficiently than increases in cortical thickness—resulting in higher levels of cognitive ability [20,21]. On the other hand, although cortical surface area is more strongly correlated with volume than thickness, thickness contributes more to the age-related decline in cortical volume [15,16] and may reflect age-related neuronal deterioration, such as decreased cell body size or synaptic connections between neurons [22]. Thus, thickness may be related to individual differences in cognition as a result of age-related structural changes, whereas cortical surface area may be related to cognition through preexisting genetic differences. Indeed, cortical thickness is most related to cognition in older as opposed to younger adults (presumably due to age-related deterioration and increased variance with age across samples; [23–25]).

In regards to the organization of these age-related changes in cortical thickness across the cortex, the currently accepted view is that age-related cortical thinning largely falls within age- and cognitively-related functional networks, rather than traditional theories based on structural anatomical locations (e.g., “frontal lobe hypothesis”; [26,27]). One study observed widespread cortical thinning most pronounced within regions implicated in the default-mode network [26], a functional network sensitive to age-related functional integrity [2–7]. Another study compared the relationship of cortical thickness of older- and middle-aged adults to young adults in four broad, functionally defined cortical areas (two associative networks, a paralimbic network, and a sensory network) and found that cortical thinning was not confined to the hypothesized associative cortical areas, but was more widespread and global [28]. However, within these areas, functionally distinct networks exist. For example, within the broadly defined associative areas, a frontal parietal, default mode, and dorsal attention network exists, and sensory areas can be parcellated into somatomotor and visual networks [29,30]. Furthermore, individual variation in cortical thickness across regions displays a similar organization as functional networks and does not necessarily follow structural anatomy [31–37].

In regards to cognition, a few studies have interpreted whole brain, exploratory analyses in terms of functional networks after discovering associations between cortical thickness and executive function task performance. While results were discussed in terms fronto-parietal control networks, there were no explicit inferential tests to examine the validity to these interpretations [25,38]. Other post hoc functional network approaches present similar challenges for interpretation and validity [39].

Surprisingly, little research has investigated the relationship of regional cortical surface area (in addition to thickness) and cognitive ability in the same population of older adults. One study found evidence of a relationship between cortical surface area and episodic memory task performance, but this investigation was confined to *a-priori* defined regions of the medial temporal cortex with a relatively small sample [40]. In samples of young and middle-aged adults, cortical surface area, but not cortical thickness, was associated with cognition ([41,42] but see [43]). The relationship of cognitive abilities and cortical surface area, in contrast to cortical thickness, appears to have a genetic origin [41] and stability throughout the lifespan [24,44]. Given this lack of understanding about the meaning of surface area and thickness, one aim of the current study was to investigate both the relationship of surface area and thickness to age-related cognition in older adults.

Additionally, functional networks are most commonly characterized as a set of functionally coupled but distributed regions [29,30]. A set of individual regions within functional networks may be important to the integrity of a functional network or overall system of brain underlying cognition (i.e., ‘hubs’ [45]). In previous studies, interpretations argue for the importance of functional networks based on the location of individual regions within a network. But, ultimately, the average efficiency of these individual regions may determine the integrity of a functional network and its role in cognition [46]. Thus, focusing analyses on the average structural integrity of important regions within functional networks, rather than focusing exclusively on individual clusters, may be a more effective means to understand structural integrity consistent with the concept of the brain as a system of interconnected regions.

In the current investigation, we address these methodological and theoretical challenges by quantifying the predictive ability and spatial extent (i.e., number of significant regions) of cortical structure of regions significantly related to cognition within individual functional networks. First, in a fully cross-validated framework, we develop predictive models by selecting local regions significantly associated with these cognitive abilities, averaging the cortical morphometry of the selected regions together, and fitting a linear regression model to the average structural quantities (i.e., a single beta coefficient weight) to predict cognitive abilities (for a similar approach with functional connectivity, see [47]). In a separate set of analyses, we quantified the number of regions robustly related to these cognitive abilities within each network to determine the spatial extent of this relationship. Furthermore, how cortical thickness mediates the effects of age on cognition may provide clues as to how brain structure *influences* cognitive function through age related processes [14]. Thus, we also determined how cortical structure mediates the age-cognition relationship in each of these analyses to provide evidence of age-related processes.

We combine performance on multiple laboratory and neuropsychological tasks to define two cognitive constructs that robustly decline with age—executive function and episodic memory. These two cognitive abilities are thought to share common and distinct processes in cognitive aging and have well-known neurobiological underpinnings [48,49].

We hypothesized that default mode network cortical structure may be particularly important for relationships with episodic memory ability, given its well established role in age-related functional declines[2–7], its hypothesized role in age-related structural declines [26], and its importance in episodic memory encoding processes [50]. However, the default mode network functional integrity has also been associated with executive function and processing speed in older adults, suggesting its role in more general cognitive ability in aging [2–4,8].

Executive function relies on the control of attentional processes; these control processes are thought to largely rely on the flexible communication of the control network with other networks [51]. Thus, the cortical structure of the control network may be particularly important in relationships with executive function ability [25]. In addition, the interplay of attentional networks (i.e., the dorsal and salience networks) is also important for efficiently selecting appropriate stimuli, a process particularly important for executive function tasks [52].

On the other hand, the primary sensory networks are often used as control regions and may not have as strong of a relationship with age and cognition as the other associative networks. In terms of functional integrity, some studies have found internetwork connectivity within the somatomotor network, along with other networks (i.e., the default mode network), to be sensitive to changes in age [6] while others have found the functional integrity within the sensorimotor and visual networks to be unrelated to age [5,7]. In terms of brain structure and cognition in older adults, two studies reported significant clusters of cortical thickness in the somatomotor cortex yet the interpretation of these clusters were not discussed or selected for further post-hoc analyses which illustrates the importance of the current analyses [25,39]. As stated previously, in regards to aging, some whole brain studies have shown widespread cortical thinning including the somatomotor and visual cortex as well [53].

## Methods

### Participants

Two hundred thirty-five community dwelling older adults were recruited. To be eligible, participants were required to be right-handed, score at least a 23 on the mini-mental state examination (MMSE), and have no MRI contraindications (e.g., metal in body, no claustrophobia). Before starting the first session, participants provided written consent. Specifically, a University of Illinois Institutional Review Board approved the study, and written informed consent was obtained from all participants and the study was performed in accordance with the 1964 Declaration of Helsinki. Participants received financial reimbursement. One hundred eighty-one participants completed three sessions of screening, neuropsychological, and neuroimaging testing. In these sessions, participants underwent two sessions of cognitive testing and one session of neuroimaging testing in a fixed session and task order.

Finally, after data collection, we screened for good MRI data quality (e.g., no evidence of motion affecting MRI processing procedures or anamolies). The final sample used for analyses included 165 participants (105 female) between 60–89 years of age (M: 69.5, SD: 6.58) with an average of 16.74 (self-reported) years of education (SD: 3.29).

### Cognitive tasks

Cognitive tasks previously used in aging research were selected to examine a variety of cognitive abilities falling under domains implicated in age-related decline including long term memory, processing speed, and executive function (see Table 1 for descriptive statistics for each task).

**Table 1:** Descriptive statistics of individual tasks and cortical structure.

Variable	Mean (SD)
**Mini Mental State Examination (MMSE)**	28.71 (1.36)
Flanker Incongruent (milliseconds)	844.57 (123.52)
Trail Making Part B (seconds)	77.67 (24.74)
Spatial Working Memory (accuracy)	0.79 (0.12)
Letter N-Back (2 Back)	0.82 (0.15)
Category Fluency (Animals + Vegetables)	40.75 (8.9)
Face-Scene (D’)	0.86 (0.59)
Spatial Reconstruction (Swaps)	0.13 (0.07)
Dot Comparison (milliseconds)	2634.02 (688.7)
Digit Symbol Coding	63.06 (12.43)
CVLT Free Recall (Delayed)	11.18 (3.38)
Story Free Recall (Delayed)	11.37 (3.32)
Total surface area (millimeters^2^)	157614.2 (15775.83)
Average thickness (millimeters)	2.39 (0.12)

*Note*. The MMSE was used to screen for cognitive impairment and inclusion in the study. Total surface area and average thickness are summary metrics of entire cortex

To create composite cognitive ability scores, we used a data-driven approach. That is, we ran a principal component analysis with a varimax rotation. We retained the first two components as they occurred before the inflexion point (see Table 1; [54]). Although one might expect processing speed to emerge as an independent component [55], the first component grouped perceptual speed and executive function together. However, the processing speed tasks used in the current study can also be conceptualized as executive function tasks given the high demand on comparison processes (see [56]) and share a tight link with more traditional executive function tasks [57,58]. Thus, the first component appeared to capture “common” executive function ability as conceptualized elsewhere [59]. The second principal component largely agreed with previous work on episodic memory using a subset of the current sample [60] with the exception of Category Fluency, which loaded most highly on the episodic memory component despite traditionally being thought of as an executive function task. This could be due to processes or strategies involving retrieval from long-term memory [61]. For each component, we averaged standardized scores for tasks that demonstrated the higher loading onto that component compared with the other component (no loading was below .5 for a task’s selected component with the exception of N-Back with a loading of .46 on the Executive Function component; Table 2). To attenuate the influence of outliers, we winsorized cognitive task performance before running the principal component analysis and creating cognitive ability scores to control for outliers (for individual scores falling outside of 3 standard deviations of the mean). We identified one outlier for Flanker and Logical Memory task performance, two outliers for Trail Making, three outliers for Dot Comparison and NBack task performance, and four outliers for Spatial Working Memory task performance. Below are brief descriptions of each task for each cognitive ability (i.e., component from the PCA).

**Table 2:** Principal component analysis loadings after varimax rotation.

Task	Component 1: Executive Function	Component 2: Episodic Memory
Flanker Incongruent	**0.59**	0.04
Trail Making Part B	**0.71**	0.19
Spatial Working Memory	**0.62**	0.21
Letter N-back (2 Back)	**0.52**	0.14
Category Fluency (Animals + Vegetables)	0.27	**0.56**
Face-Scene (D’)	0.17	**0.58**
Spatial Reconstruction (Swaps)	0.13	**0.69**
Dot Comparison	**0.74**	0.09
Digit Symbol Coding	**0.69**	0.31
CVLT Free Recall (Delayed)	0.22	**0.74**
Story Free Recall (Delayed)	0.04	**0.68**
% Variance explained	0.25	0.21

#### Executive function tasks

*Flanker* [61]. Five arrows appeared in the center of the screen with a center arrow and two flanking arrows on each side. Participants were asked to respond to the direction of the center arrow. On half the trials, the center arrow was in the same direction as the flanking arrows (congruent trials) and, on the other half, the center arrow was pointed in the opposite direction as the other arrows (incongruent trials). Average reaction time of the incongruent trials was used as the performance metric.

*Trail Making* [62]. Participants were presented with a sheet of 25 numbers distributed across a sheet of paper. In ascending order and as fast as possible, participants drew connections between these numbers without lifting their pencil (Part A). A second sheet was then presented with digits and letters. Participants were instructed to draw connections between letters and digits by alternating between the two categories in ascending order (Part B). The time taken to complete Part B (in seconds) was used as the performance metric.

*Letter N-Back* [63,64] Participants viewed a sequence of centrally presented letters. For each letter, participants were instructed to determine if the current letter matched the previous letter (first block, 1-back) or two letters back (second block, 2-back). There were five 20-letter sequences per condition for a total of 100 trials (25 target trials for all conditions and 10 lure trials for the 2-back) per condition. Mean accuracy across the 2-back condition was used as the variable of interest.

*Spatial Working Memory* [65,66]. Participants viewed a configuration of black dots on the screen. After a brief delay, a red target dot probe appeared. Participants were instructed to detect whether the red dot probe was in the same or different position as the black dots. Forty trials (20 same and 20 different) per condition were presented with dot locations varying randomly. Average accuracy across conditions was used as the performance metric.

*Digit Symbol Coding* [67]. Participants were instructed to write the symbol that corresponded to each digit amongst a list of digits. The goal was complete as many items in the list as possible within 2 minutes. Nine unique symbols corresponded to a specific digit (1–9), which was visible in a key participants were required to reference. The total number of correctly written symbols was used as the dependent measure.

*Dot Comparison* [68]. Two 4x4 matrices of dots were displayed to the left and right of fixation. Each dot was either filled or unfilled, creating a dot pattern. Participants were instructed to indicate whether the two dot patterns were the same or different. On half the trials, the dot pattern differed by one dot (one filled and one unfilled) and, on the other half, both dot patterns were the same. Performance was measured by mean response time across the experiment.

*Category Fluency*. Participants were given 1 minute to name as many instances of a category as possible. Two categories were used: fruits/vegetables and animals. The total number of unique words from both categories was used to measure performance.

#### Episodic memory tasks

*Face-Scene Relational Memory* [60]. Participants were presented with a face and scene in the background. After each face-scene trial is presented, participants were asked to indicate whether they thought the face fits with the scene. After 24 encoding trials of unique faces and scenes and a 20 second break, participants were then presented with another series of face-scene pairs. In this part, the task was to indicate whether the face-scene pair was present in the first part of the experiment. Participants were presented with 24 recognition trials. This same task was conducted with new face-scene pairs across 3 runs during the MRI session (with the fMRI results to be reported elsewhere). The probability of hits minus the probability of false alarms (d’; [69]) was used to measure memory performance.

*Spatial Reconstruction* [70]. An arrangement of unique line drawings was presented. Participants were instructed to use the mouse to click on each drawing to indicate it was studied. This study period was self-paced. Following the study period and a 4000 ms delay, the drawings were aligned at the top of an otherwise blank screen. Participants were instructed to use the mouse to click and drag them into where they thought they were positioned in the study phase. Participants completed 3 practice trials and 15 real trials. Of interest were the percentage of “swaps” participants made across the 15 trials. A swap occurred when participants switched stimuli between two locations containing stimuli in the study arrangement [60,70].

*Logical Memory Story Free Recall* [67]. Participants listened to a story and instructed to recall as much as they could remember. After a 30-minute delay, participants were asked to recall as much from this story as possible. The dependent measure was the number of story units correctly recalled in the delayed free recall.

*California Verbal Learning Test (version 2)* [71]. The experimenter read a list of 16 nouns drawn from four semantic categories. After the list was read, participants were instructed to recall as many words as possible. This procedure was performed for five consecutive trials (i.e., immediate recall trials). After a twenty-minute delay, where participants performed a problem-solving task (not part of this study), participants were instructed to recall as many words from the list as possible. The total number of words recalled after the long delay was used as the performance metric.

### Structural MRI acquisition and processing

All imaging was performed on a 3T Siemens Trio MRI system with a 12-channel head coil. For each imaging session, high resolution T1-weighted anatomical images were collected using a MPRAGE (Magnetization Prepared Rapid Gradient Echo) protocol (192 slices, GRAPPA acceleration factor of 2, voxel size = .9 x .9 x .9 mm, TR = 1900 ms, TI = 900 ms, TE = 2.32 ms, flip angle = 9°, FoV = 230 mm).

Each participant’s T1 structural volume was processed through Freesurfer version 5.3 (http://surfer.nmr.mgh.harvard.edu/; [72]). In summary, a surface reconstruction of the white matter/gray matter boundary and the cortical (pial) surface were created through non-brain tissue removal, Talaraich transformation, intensity normalization, segmentation of the grey/white matter boundary, and tessellation. Each reconstruction was visually checked for plausibility of the reconstruction and major topological inaccuracies were corrected with the recommended intervention procedures and reprocessed (i.e., white and pial surface edits and control points).

For manual corrections, trained operators corrected points suggested for manual intervention including talairach registration, white matter intensity correction via control points, and removal of non-brain tissue (i.e., dura) affecting the initial surface reconstruction. To ensure consistency across operators, each operator was trained using a set of common intervention strategies taken from the freesurfer tutorial (http://surfer.nmr.mgh.harvard.edu/fswiki/FsTutorial/) and in-house examples. Minor manual realignment of talaraich registrations to optimize alignment of anatomical landmarks (e.g., slight realignment of registration to optimize match of the corpus callosum outline) were reported on approximately 80% of participants. The addition of at least one control point was reported on 23% of participants. Minor deletions of non-brain tissue affecting cortical surface estimates were reported on approximately 70% of participants.

Important to the current analyses, the surface area and thickness of each individual vertex was quantified. These quantities were smoothed with a 10 mm full-width half maximum Gaussian kernel across the surface. Finally, surface reconstructions were then transformed to a common spherical coordinate system based on cortical folding patterns.

**Fig 1:**
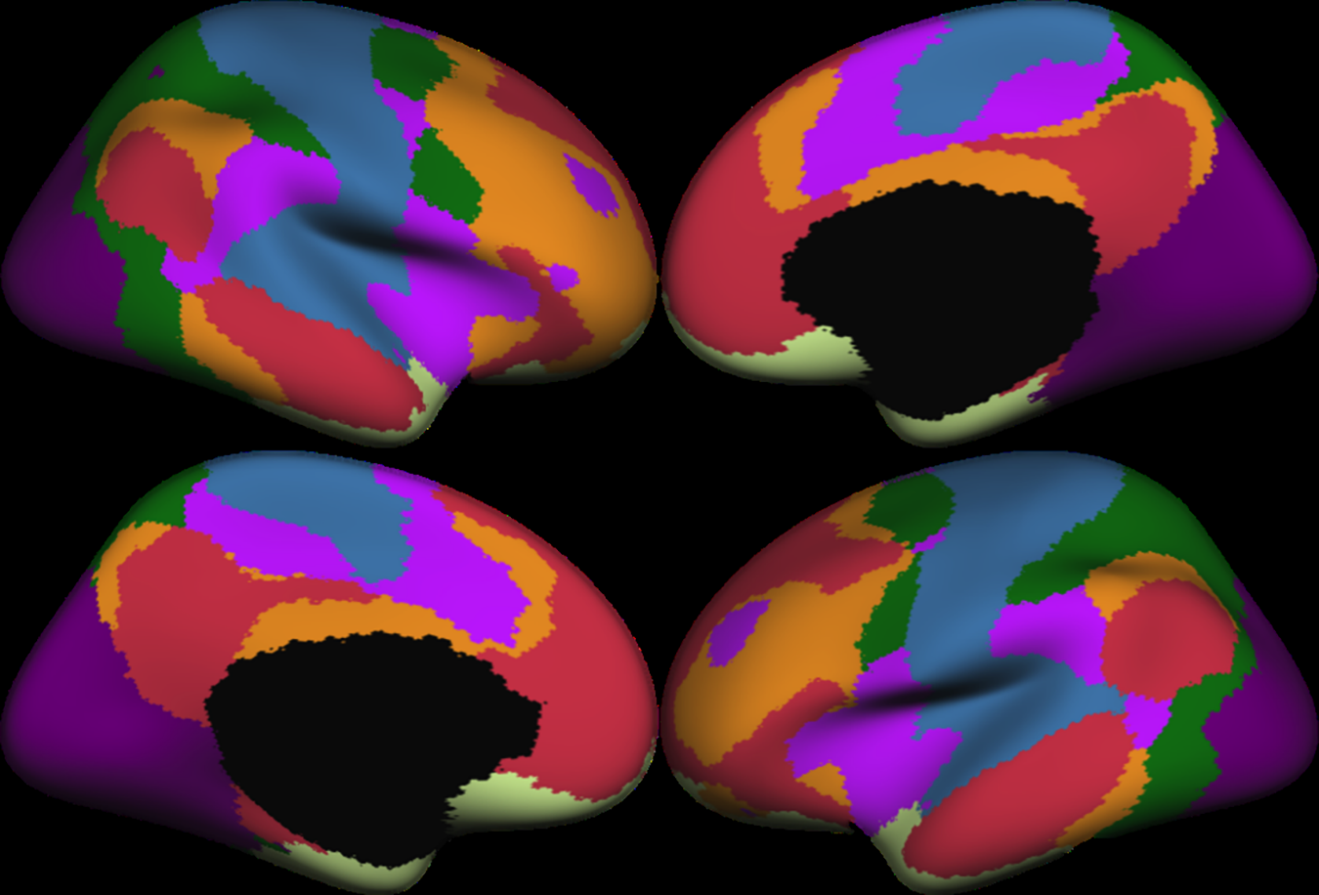
The functional network parcellations used in the current study from [29]. The network colors correspond to the colors used in the original paper (network colors: visual = violet, somatomotor = blue, dorsal attention = green, salience = fuchsia, limbic = cream, control = orange, default mode = red)). Note, the black area represents medial areas (e.g., subcortex) not included in surface-based analyses.

The 7-network cortical parcellation created from a previous study’s Freesurfer surface-based functional connectivity analysis ([29]; Fig 1) was used to assign each vertex to a network. This network parcellation was chosen given its creation with Freesurfer, its smaller number of comparisons between networks (compared to a 17 network parcellation also available) in inferential testing and convergence with other popular volume-based network parcellation schemes [30]. Additionally, this 7-network parcellation has shown sensitivity to age-related effects in terms of functional connectivity in a previous study and used the same cortical surface-based registration procedure (i.e., Freesurfer) as the current study to define cortical functional networks [6].

For each subject, Freesurfer-formatted surface files containing the areal quantities of each vertex (for both hemispheres) were imported into python using nibabel (see www.nipy.org/nibabel; version 2.1.0) and concatenated using pandas library tools [73] creating a matrix of 165 rows (number of participants) and 299374 columns (each representing a morphometric estimation of individual vertices from surface models assigned to one of the network labels). Seven individual network matrices were created by filtering vertices assigned to an individual network. The whole brain and individual network matrices were used in the predictive modelling pipeline and subsequent analyses detailed below.

### Cognitive ability prediction analyses

**Fig 2:**
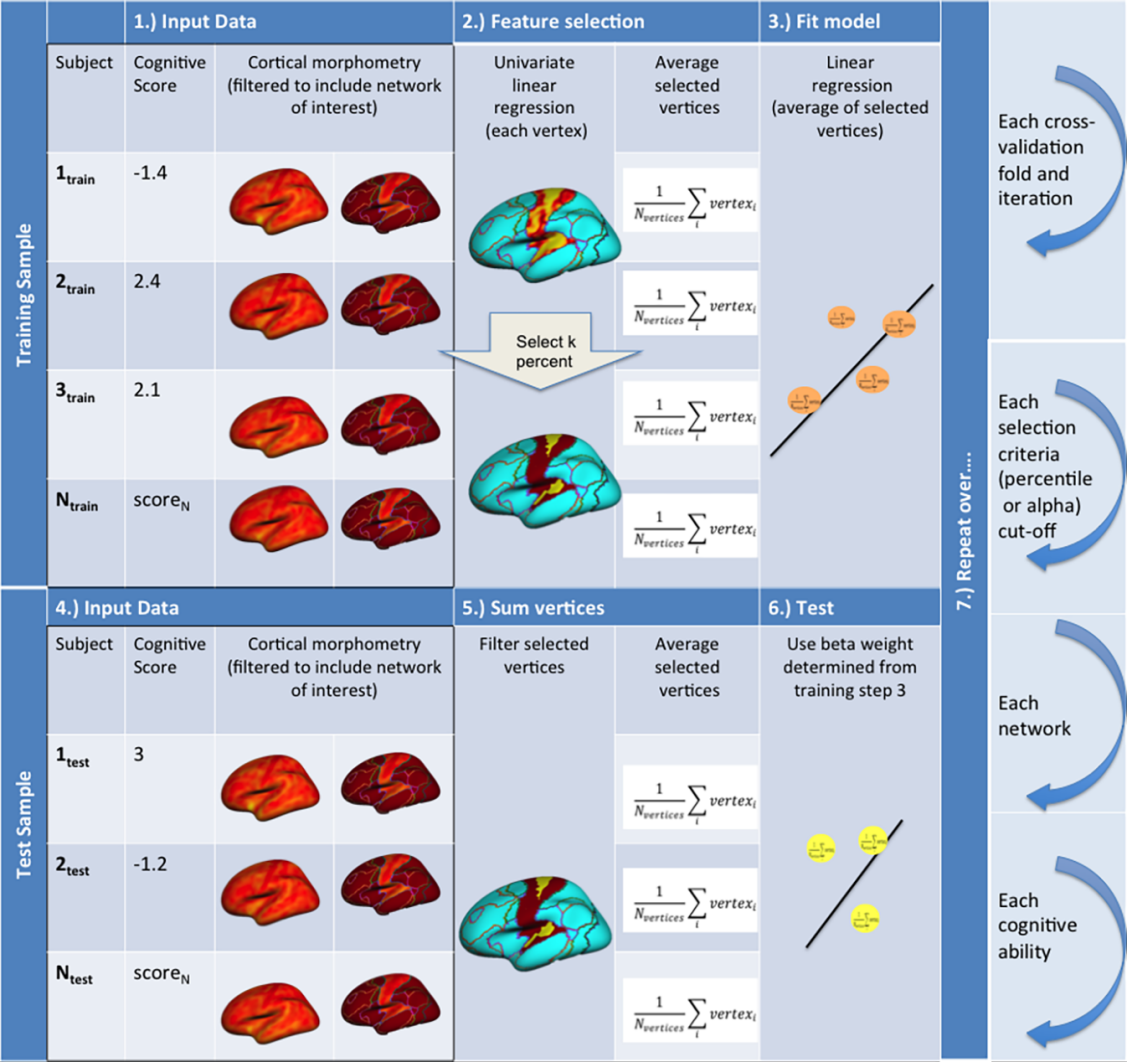
A schematic of the cross validation workflow for prediction analyses. The workflow is displayed as 7 steps with steps 1–6 describing the model train and test workflow within each cross validation fold.

For a schematic of the cross-validation prediction analysis workflow, see Fig 2. A repeated five-fold cross validation procedure was used for training and testing [74]. That is, 100 random sets of 5-fold training and testing samples of participants were obtained for the predictive modeling workflow detailed below.

Within each fold’s training sample, each vertex measure (i.e., point-wise local surface area or thickness values) was scaled using statistics robust to outliers (i.e., centering on the median and scaling with interquartile range between 25% and 75%). Then, the univariate linear relationship for each of these scaled vertex measures with cognitive ability (i.e., target) was calculated. Vertices with p-values equal to or less than a threshold (i.e., .05, .01, .001, .0005, .0001) were selected. We used a range of these thresholds common in similar predictive modeling paradigms [47,75,76] and previous exploratory surface-based morphometry analyses [25,27]. For each individual in the training set, the morphometry of these vertices were averaged together and used to fit a linear model (i.e., a single beta coefficient weight) using the cognitive ability scores as targets. This model was then used to predict the unseen testing sample participants’ cognitive abilities (i.e., selected vertices were averaged together and fit to the training model).

Test set predictions for each participant were averaged together across folds and iterations to obtain a single cognitive ability prediction for each participant. For each cognitive ability, we obtained 5 average predicted values for each subject corresponding to each false discovery rate threshold selection criteria predictions. For inferential testing, we averaged these 5 predicted values across the 100 iterations for each participant in order to decrease multiple comparisons and for ease of interpretation for subsequent analyses.

#### Predicting cognition

We first assessed the relationship of predicted and actual cognitive scores after accounting for sex given its known relationship with morphometry [77]. We obtained a distribution of correlation coefficients after accounting for sex across 5000 samples using the bootstrap procedure [78]. We report the 5% and 95% percentile confidence intervals and the proportion of bootstrapped coefficients greater than zero to assess significance. This bootstrapped procedure was performed for each feature selection criteria (alpha thresholds) for each network and cognitive ability but inferences were drawn from the average prediction values. Note, that predictive models can be worse than chance, which is indicated by *r* < 0. This is an important distinction for traditional correlation metrics where *r* < 0 means there could be important variability between two variables.

#### Mediation of age and cognition

Whether the predictive ability of cortical thickness of the different functional networks mediates the relationship between age and cognition is an important question for understanding how structure influences the effect of age-related cognitive processes [10,14,55]. To answer this question, we computed the mediation effect in a series of mediation analyses using sex as a covariate [79]. In these analyses, we fit two models: (1) predicting cognition with only age and sex and (2) predicting cognition with age, cortical morphometry-based predictions, and sex. We compute the difference in the beta coefficients of age between the two models as a measure of the mediation effect [79]. This mediation effect was computed over 5000 bootstrapped samples. 95% bootstrap percentile CIs and the proportion of mediation effects greater than 0 were used to assess significance of the mediation effect. Age was transformed into negative values (e.g., 67 years old was transformed to -67) so all meaningful effects were positive.

### Exploratory univariate whole brain analysis

To determine the spatial extent of the relationship of regional morphometry and each cognitive measure, we calculated the correlation between (actual) morphometry of each vertex and cognitive ability scores and the mediation effect of the age-cognition relationship [79] after accounting for sex. This mediation analysis was computed in the same way as previously described for the predictive model analyses with the exception that observed cortical morphometric values (with a separate analysis for each vertex) were used as predictors rather than the prediction values. We performed these analyses across 5000 bootstrapped samples for each morphometric measure (surface area and thickness) and cognitive ability [78]. For each vertex, we calculated a bootstrap ratio (BSR) of the average bootstrap correlation divided by the standard error of the bootstrapped correlation across samples (see [80,81] for another application of the BSR in the context of linear models using brain imaging data). We then thresholded each vertex’s BSR at a range of values corresponding to *p*-values of 0.05, 0.01, 0.001, and 0.0001 (z = 1.96, 2.58, 3.3 and 3.9). A count of surviving vertices for each network was then calculated.

To infer the importance of networks in analyses, we shuffled individual surface vertex BSRs and ran the above procedure to create a “shuffled distribution” (i.e., null distribution) across 100,000 permutations. The proportion of these permutations greater than or equal to the empirical number of significant vertices was used to assess significance.

## Results

### Predictive models

Females had significantly higher episodic memory ability scores than males (*t* = 3.74, *p* < 0.001, *95% CI* [0.18 0.6 ]). No sex differences were detected for executive function (*t* = -0.28, *p* = 0.78, *95% CI* [-0.23 0.17 ]) or age (*t* = -1.21, *p* = 0.229, *95% CI* [-3.51 0.85 ]). However, in terms of average morphometry across the whole cortex, females had a thicker cortex (*t* = 3.84, *p* < 0.001, *95% CI* [0.03 0.1 ]) but smaller surface area (*t* = -7.66, *p* < 0.001, *95% CI* [-22185.12–13055.61 ]) compared to males. Given these differences, we controlled for sex in all analyses.

Participants’ cognitive ability composite scores were significantly related to age for both episodic memory (*r* = -0.36, *p* < 0.001, *95% CI* [-0.45–0.26 ]) and executive function (*r* = -0.3, *p* < 0.001, *95% CI* [-0.42–0.18 ]) showing the composite scores for the current study did indeed capture age-related variance.

#### Predicting cognition

Average cortical thickness (observed) across the entire cortex was significantly associated with executive function (*r* = 0.29, *p* < .001, *95% CI* [0.19 0.37 ]) and memory (*r* = 0.22, *p* = 0.01, *95% CI* [0.09 0.34 ]). Total surface area across the entire cortex was not associated with either cognitive ability (Memory: *r* = 0.06, *p* = 0.323, *95% CI* [-0.04 0.17 ]; Executive Function: *r* = 0.06, *p* = 0.436, *95% CI* [-0.08 0.2 ]).

**Fig 3:**
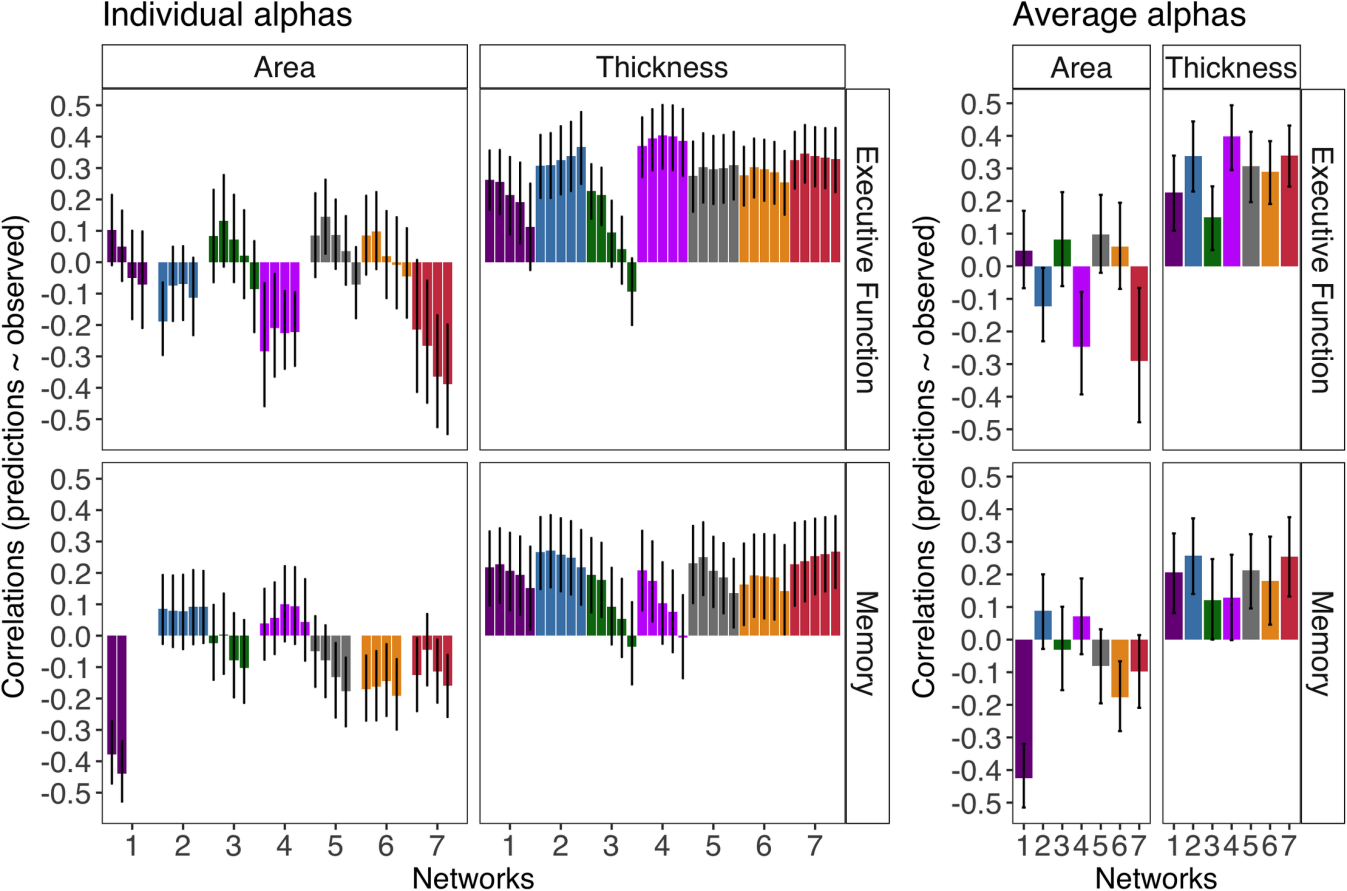
The correlation of cortical morphometry-based cognitive predictions and actual cognitive predictions after accounting for sex (i.e., predictive ability) with 95% bootstrapped CIs for surface area and thickness. For individual p-value thresholds (left), most liberal to most conservative thresholds are displayed from left to right (.05,.01,.001,.0005,.0001). The averaged predictions across different alpha thresholds were used for inferences (right). Blank spaces indicate models with no vertices selected for an alpha threshold in any fold and iteration. The networks are color coded and ordered according to the Yeo et al., (2011) scheme (visual = 1 (violet), somatomotor = 2 (blue), dorsal attention = 3 (green), salience = 4 (fuchsia), limbic = 5 (cream), control = 6 (orange), default mode = 7 (red)).

**Fig 4:**
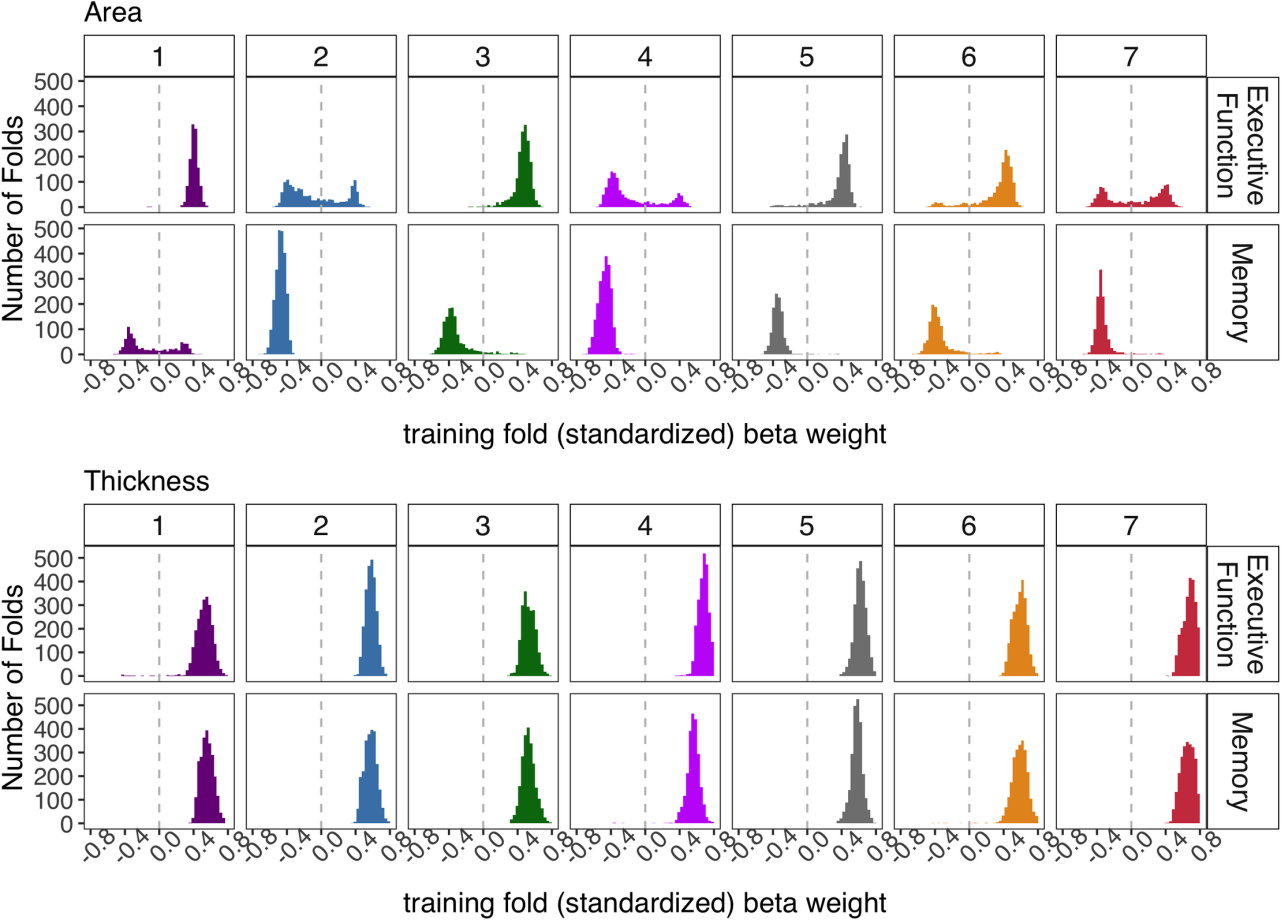
Distribution of standardized beta weights across all 500 fold training sets (5 folds, 100 iterations) and across all alpha thresholds (.05,.01,.001,.0005,.0001) for each model type. Note, these are the standardized beta weights of the average of the selected regional morphometry. The networks are color coded and ordered according to the Yeo et al., (2011) scheme (visual = 1 (violet), somatomotor = 2 (blue), dorsal attention = 3 (green), salience = 4 (fuchsia), limbic = 5 (cream), control = 6 (orange), default mode = 7 (red)).

As displayed in Fig 3, cortical thickness (the average across thresholds), but not cortical surface area, significantly predicted executive function and memory for all individual networks (*p*s < 0.001). For some individual networks, the predictive ability of surface area was significantly worse than chance for executive function (salience and default mode networks; ps < 0.001) and memory (visual and control networks; *p*s < 0.001). Fig 4 reveals the training beta weights of these models have a bimodal distribution across folds and selected thresholds centered around 0 with negative and positive weights (this is apparent for the visual network for memory and the salience and default mode network for executive function). The distribution of these below-chance model weights indicates a low reliability. In these models, across many of the validation folds at more conservative thresholds, few or no vertices were selected (Figure B in S1 File), which also speaks to the weak relationship of surface area and cognition. However, there was at least one p-value threshold with some vertices selected for all folds (e.g., the *p* = 0.05 threshold had 0% of folds with no vertices selected for all networks; Figure B in S1 File). This makes inferences about any network (or lack thereof) not biased by a fewer number of folds (Figure A in S1 File).

Given the significant relationships of average cortical thickness across the entire cortex to both cognitive abilities, we evaluated whether the predictive ability of network thickness-based models remained significant after accounting for average thickness. For executive function, the predictive ability of cortical thickness of the somatomotor network (*r* = 0.08, _*p*_ = 0.036, _95% CI_ [0.02 0.15 ]), the salience network (*r* = 0.17, *p* < .001, *95% CI* [0.1 0.23 ]), the limbic network (*r* = 0.12, *p* = 0.028, *95% CI* [0.03 0.2 ]), and the default mode network (*r* = 0.09, *p* = 0.01, *95% CI* [0.03 0.14 ]) remained significant. For memory, the predictive ability of cortical thickness remained marginally significant for the limbic network (*r* = 0.11, *p* = 0.074, *95% CI* [0.01 0.2 ]) and default mode networks (*r* = 0.05, *p* = 0.056, *95% CI* [0.01 0.1 ]).

#### Relationship with age

The cortical thickness-based predictions of cognition (memory and executive function) were significantly related to age for all 7 networks with *r*s ranging from 0.25 to 0.38, all *p*s < 0.001).

Furthermore, average cortical thickness across the entire cortex was negatively associated with age (*r* = -0.32, *p* < 0.001, *95% CI* [-0.44–0.19 ]). Total surface area across the entire cortex was not significantly associated with age (*r* = -0.07, *p* = 1.741, *95% CI* [-0.18 0.03]).

Next, we examined if the significant predictive abilities of thickness remained after accounting for average cortical thickness across the entire cortex. We found cortical thickness-based predictions of executive function were related to age for the visual (*r* = 0.1, *p* = 0.023, *95% CI* [0.03 0.19 ]), somatomotor (*r* = 0.1, *p* = 0.016, *95% CI* [0.03 0.16 ]), salience (*r* = 0.12, *p* = 0.007, *95% CI* [0.04 0.18 ]), and limbic (*r* = 0.15, *p* = 0.018, *95% CI* [0.05 0.25 ]) networks with a marginally significant relationship with the default mode network (*r* = 0.06, *p* = 0.07, *95% CI* [0.01 0.12 ]). For the cortical thickness-based predictions of memory, only the salience (*r* = 0.07, *p* = 0.033, *95% CI* [0.02 0.13 ]) and limbic networks (*r* = 0.18, *p* = 0.019, *95% CI* [0.05 0.3 ]) showed significant relationships with age.

#### Mediation of age and cognition

**Fig 5:**
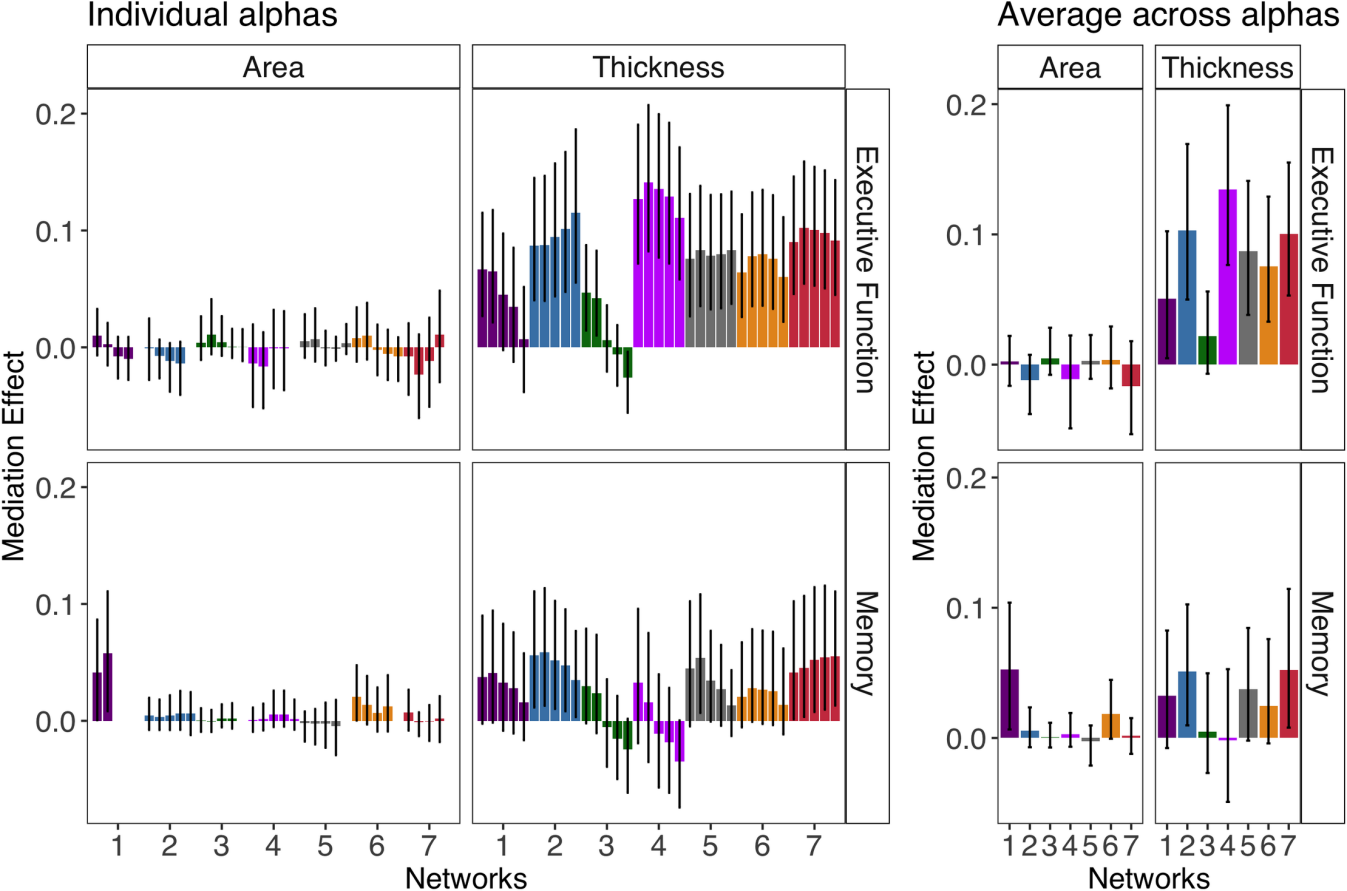
The mediation effect estimates (with 95% bootstrapped CIs) of cortical morphometry-based cognitive predictions from mediation models using sex as a covariate. For individual thresholds (left), most liberal to most conservative thresholds are displayed from left to right (0.05, 0.01, 0.001, 0.0005, 0.0001). Blank spaces indicate models with no vertices selected for an alpha threshold in any fold and iteration. The averaged predictions across different alpha thresholds were used for inferences (right). The networks are color coded and ordered according to the scheme in [29] (visual = 1 (violet), somatomotor = 2 (blue), dorsal attention = 3 (green), salience = 4 (fuchsia), limbic = 5 (cream), control = 6 (orange), default mode = 7 (red)).

Across all mediation models, all direct effects were significant, showing that age was significantly related to cognition independent of cortical thickness-based predictions (*p*s < .001). Fig 5 shows the mediation effects for both thickness-based and surface area-based predictions (although not all networks had a significant effect of both cognition and age, all effects are displayed here for completeness). The somatomotor and default mode network thickness-based predictions were the only two networks to significantly mediate the age and cognition relationship for both executive function (default mode network: *r* = 0.1, *p* < 0.001, *95% CI* [0.05 0.16]; somatomotor network: *r* = 0.1, *p* < 0.001, *95% CI* [0.05 0.17 ]) and memory (default mode network: *r* = 0.05, *p* = 0.04, *95% CI* [0.01 0.11 ]; somatomotor network: *r* = 0.05, *p* = 0.034, *95% CI* [0.01 0.1 ]). The thickness-based predictions of the visual, salience, limbic and control networks significantly mediated the relationship of age and executive function but not memory (*p*s < 0.001).

For surface area, no interpretable and significant mediation effects existed. Visual network surface area-based predictions showed a mediation effect but this was driven by significantly below chance accuracy for relationships with cognition and age.

Given the significant association of average cortical thickness across the entire cortex to both age and cognitive ability scores, we tested whether cortex-wide average thickness mediated the age-cognition relationship. Average thickness showed a significant mediation effect for executive function (*r* = 0.07, *p* < .001, *95% CI* [0.03 0.12 ]) but not memory (*r* = 0.04, *p* = 0.116, *95% CI* [0 0.1 ]).

After accounting for average thickness, only the salience network remained as a significant mediator of the relationship between age and executive function (*r* = 0.06, *p* = 0.008, *95% CI* [0.02 0.1 ]). No analyses reached significance for mediation effects on the age and memory relationship after accounting for average thickness (*p*s > 0.05).

### Exploratory whole brain analysis of the relationship between cortical morphometry and cognition

#### Relationship with cognition

Irrespective of functional network, thickness had many more total significant regional associations (based on the bootstrapped ratio scores (BSR)) with both cognitive abilities than surface area for each threshold. For executive function, the total number of significant vertices ranged from 117432 (39.23%) for thickness and 38932 (13%) for surface area for the BSR = 1.96 threshold to 9791 (3.27%) for thickness and 9791 (3.27%) for surface area for the BSR = 3.9 threshold. For memory, the total number of significant vertices ranged from 89493 (29.89%) for thickness and 23784 (7.94%) for surface area for the BSR = 1.96 threshold to 4018 (1.34%) for thickness and 78 (0.03%) for surface area for the BSR = 3.9 threshold.

**Fig 6:**
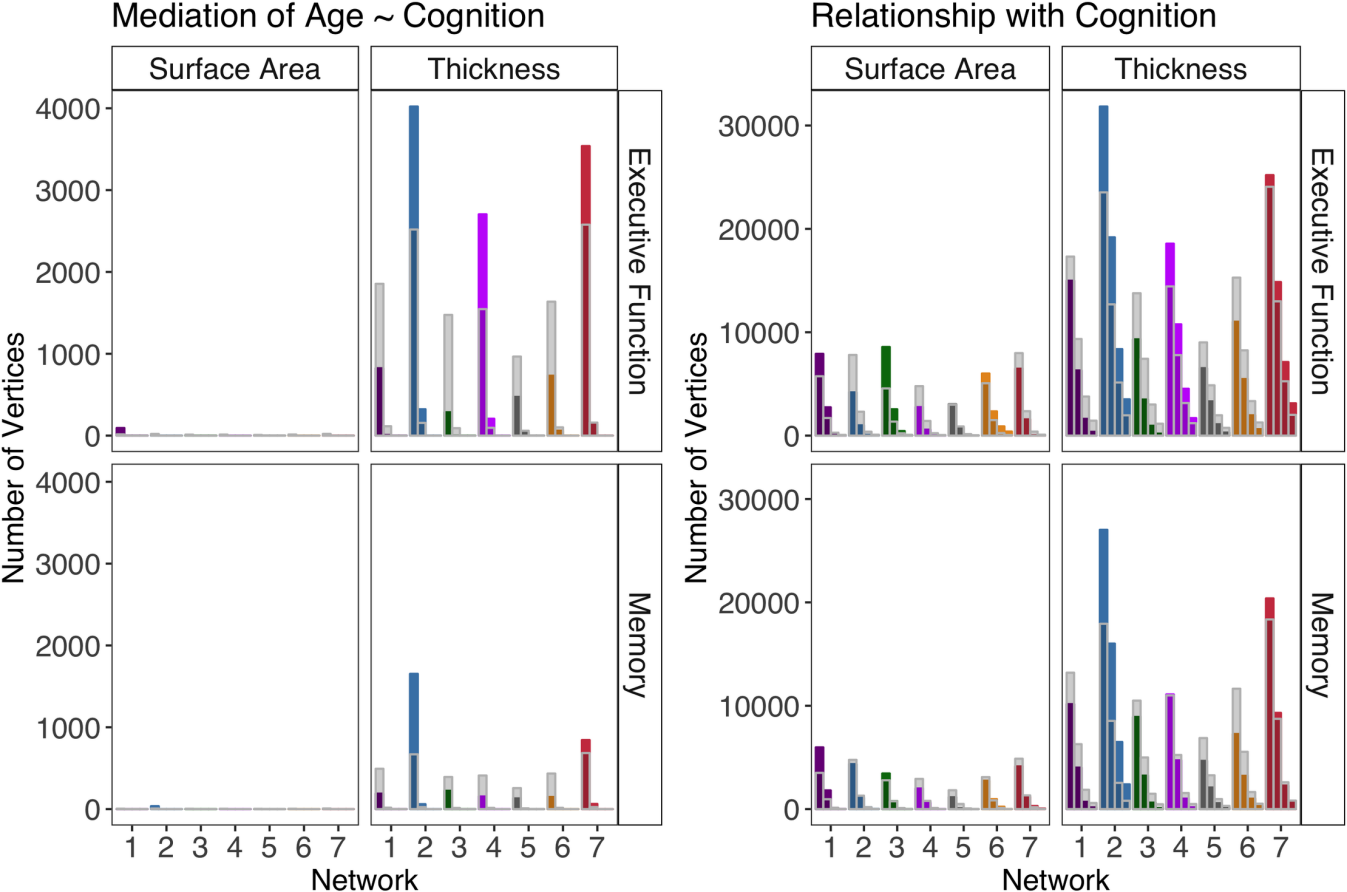
**Number of individual vertices selected within each network based on bootstrap ratio for BSR thresholds (from left to right) of 1.96, 2.58, 3.3 and 3.9**. The translucent grey bars superimposed on the network-colored bars show the average number of significant regions by chance (i.e., average significant vertices across 100000 permutations of network labels). The networks are color coded and ordered according to the scheme in [29] (visual = 1 (violet), somatomotor = 2 (blue), dorsal attention = 3 (green), salience = 4 (fuchsia), limbic = 5 (cream), control = 6 (orange), default mode = 7 (red)).

Fig 6 (right) contains the number of significant vertices for each network compared to chance level for each network (represented by the translucent grey regions superimposed on each network-colored bar). The relationship of cortical thickness of the default mode and somatomotor network to both memory and executive function had a significantly greater number of vertices that survived the chosen BSR thresholds of 1.96 (*p* < 0.05), 2.58 (*p* < 0.01), 3.3 (*p* < 0.001) and 3.9 (*p* < 0.0001) than chance (Fig 6). The cortical thickness of the salience network had a significantly greater number of vertices surviving all thresholds for executive function (*p* < .001) but significantly less than chance for memory (*p* < 0.001; Fig 5). All other networks were either significantly less than chance or did not differ significantly from chance for all thresholds (Fig 6). For surface area, the dorsal attention and control networks, as well as the visual network, had more significant vertices than chance across thresholds in its association with executive function (*p’s* < 0.001)). For memory, the visual and dorsal attention networks contained a total number of vertices greater than chance.

**Fig 7:**
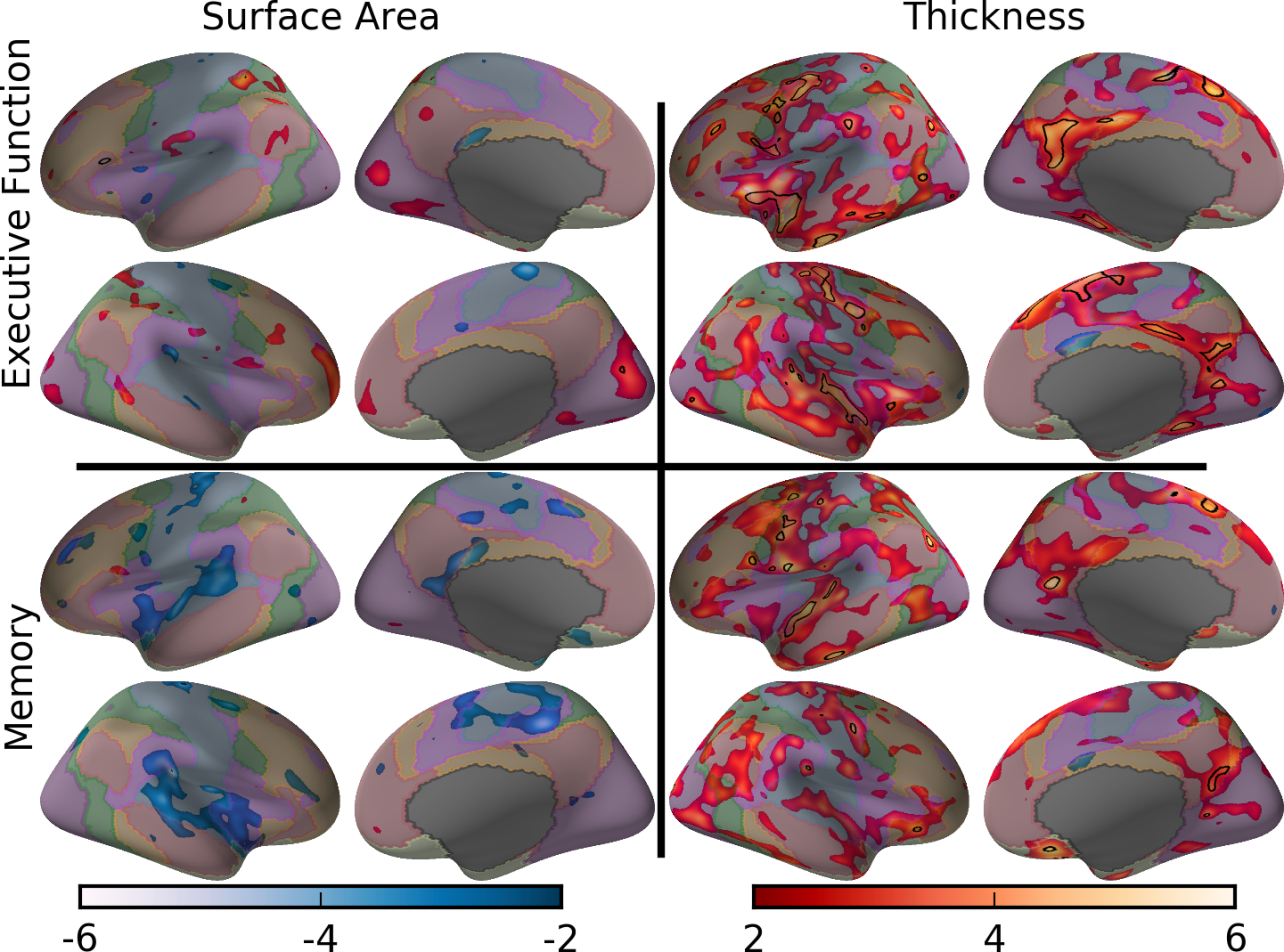
Bootstrap ratios across the cortical overlaid on the color-coded functional network boundaries. The black outlines surround regions displaying a significant mediation effect. For visualization purposes, displayed in these Figs is the BSR = 3.3 (p < 0.001) threshold for the relationship of cognition and morphometry and BSR = 1.96 (p < 0.05) for the mediation effect of morphometry on the age-cognition relationship. For the relationship of age and morphometry, see Figure E in S1 File.

Displayed in Fig 7 is a visualization of clusters above the 3.3 BSR threshold. Figure C in S1 File contains the unthresholded spatial map of regression analyses with network boundary labels.

#### Mediation of the age-cognition relationship

We also investigated the extent of the mediation effect on the age-cognition relationship. For total significant vertices, cortex thickness had many more regionally significant vertices than surface area for each cognitive ability (Executive Function: 12575 (4.2%) for thickness and 102 (0.03%) for surface area at the BSR = 1.96 threshold and 776 (0.26%) for thickness and 0 (0%) for surface area at the BSR = 2.58 threshold; Memory: 3343 (1.12%) for thickness and 36 (0.01%) for surface area at the BSR = 1.96 threshold and 123 (0.04%) for thickness and 0 (0%) for surface area at the BSR = 2.58 threshold). No significant vertices emerged for all mediation analyses at the more conservative thresholds (BSRs of 3.3 and 3.9).

Fig 6 (left) contains the number of significant vertices for each network compared to chance level for each network (i.e., shaded regions in each bar). As in the relationship with cognition, the somatomotor and default mode networks had a total number of vertices with significant mediation effects above chance and the salience network contained a total number of vertices above chance only for executive function (*p’s* < 0.001; Fig 6) at thresholds containing significant vertices (BSRs of 1.96 and 2.58). However, for surface area, only the visual attention network for executive function and the somatomotor cortex for memory at the lowest threshold (BSR = 1.96) had any significant vertices (Fig 6).

In Fig 7, the black outlines circumscribe regions of cortex containing significant mediation effects at a BSR of 1.96. Figure D in S1 File contains the unthresholded spatial map of the mediation analyses with network boundary labels. Figure F in S1 File shows the observed bivariate correlation (*r)* values for all variables involved in the previous exploratory whole brain analyses as well as cortical volume (i.e., product of surface area and thickness).

We also included the spatial maps of the associations between regional thickness, surface area, and volume in Figure G in S1 File. As expected, regional surface area exhibited a stronger relationship with volume than thickness [18]. Furthermore, surface area and thickness appeared to exhibit a weaker, negative relationship across the cortex [18].

## Discussion

One emerging view in the cognitive neuroscience of aging is that cortical structural declines align with a functional network organization. In the current study, we investigated to what extent the cortical structure of 7 major functional networks predicts executive function and episodic memory in older adults. To better attribute these structural relationships to age-related processes, we also investigated cortical structure’s mediating role in the age-cognition relationship. We showed cognitive function-specific and shared patterns of cortical structure within the 7 functional networks. Most importantly, this investigation introduces an approach to use organizing principles from functional networks and distinct cortical morphometric properties in the ongoing search to understand the relationship between structure, cognition, and age in older adults.

### Implications for the selection of cortical morphometric phenotype in older adults

In terms of cortical morphometric phenotypes, cortical thickness, but not surface area, displayed a widespread relationship with cognitive ability. The thickness of all individual functional networks (and global thickness) predicted cognitive abilities and age, and was much more spatially expansive than surface measures (i.e., had a much higher number of significant regions associated with cognition and that mediated the age-cognition relationship).

In younger adults, cortical surface area, in contrast to thickness, has shown a stronger relationship with cognition [41,42] but see [43] and has exhibited a genetic origin [41]. However, cortical thickness, to a greater extent than surface area, has been found to contribute to age-related decline in cortical volume [15]. This cortical thinning appears to accelerate at the onset of what is classified as an older adult (around 60 years of age [15]). Therefore, individual differences in cortical thickness, rather than surface area, may be more predictive of cognition in older adult samples as a result of age-related processes (but see [40]). In the current study, this is supported by the mediation effect of thickness, but not surface area, on the age and cognition relationship Furthermore, neuronal structural complexity, rather than a loss of neuronal number, may partially underlie brain volume decline in normal aging [22]. Cortical surface area is thought to mark neuronal number [19], whereas cortical thickness is hypothesized to mark neuronal structural complexity, although this has not been empirically confirmed [82]. Aging leads to a reduction in neuronal complexity through a reduction in the number of dendritic spines and synapses and reduced intracortical myelination, rather than a reduction in neuronal number [22,82]. The results of the current study provide potential support for cortical thickness as an index of these age-related changes in brain structure. However, given the cross-sectional nature of the current study, future work should apply this network-based approach in longitudinal designs and intervention-based studies proven to change cortical structure [83].

### Network- and cognitive- specificity of cortical thickness, age and cognition relationships

While cortical thickness of most networks predicted memory and executive function, only the default mode and somatomotor cortex significantly mediated the relationship between age and both cognitive abilities. Similarly, for both cognitive abilities, we found thickness in a greater number of regions located within these two networks to be associated with cognition and to mediate the age-cognition relationship. Taken together, this shows a unique role for these networks in age-related general cognitive ability.

The current study is consistent with the role of the default mode network’s structural and functional integrity in aging and cognition [27,39]. The default mode network cortical thickness may be particularly important for relationships with episodic memory ability, given its well established role in age-related functional declines [2,3,5–7], its hypothesized role in age-related structural declines [26], and its importance in episodic memory encoding processes [50]. However, the default mode network functional integrity has also been associated with executive function and processing speed in older adults, suggesting its role in more general cognitive ability in aging [2–4,8]. Indeed, the disruption of executive function processes is thought of as a mechanism of episodic memory decline during normal cognitive aging [48].

The role of somatomotor network thickness in our current study is inconsistent with the “first in, last out” hypothesis of cognitive aging [53] where it is hypothesized that sensory cortical integrity is maintained much later than frontal-parietal regions, such as in the control and default mode networks. Although inconsistent theoretically, previous studies have also reported this “unexpected” finding in the relationship of age and morphometry (thickness) across the adult life span [53], suggesting the robustness of these interpretations. This study extends the robust, but unexpected, relationship to cognition and its mediating effect on the age-cognition relationship. As stated previously, in terms of brain structure and cognition in older adults, two studies reported significant clusters of cortical thickness in the somatomotor cortex yet the interpretation of these clusters were not discussed or selected for further post-hoc analyses which illustrates the importance of the current analytical approach and results [25,39].

Interestingly, salience network thickness was particularly important for executive function as it did not mediate the age-memory relationship but was the lone network to mediate the age-executive function relationship after accounting for global thickness. The role of the salience network appears to be in line with a previous whole-brain functional connectivity study [8]. One study found functional connectivity within a subnetwork of the salience network to be specifically associated with attention and processing speed task performance [84], which rely on executive functions [58]. This subnetwork largely overlapped with important regions predicting executive function ability in the current study. Indeed, salience network regions in the current study (e.g., anterior insula) have been widely implicated in domain-general attention control, thought to be largely driven by the ability to suppress irrelevant stimuli that declines with age, in addition to task set maintenance [85,86]. A large body of work has implicated aging deficits in attentional capture [87,88], which may be due to deficits in the competitive balance between stimulus-driven and goal-directed attention [52] related to the structural integrity of the salience network.

Importantly, despite the established role of the dorsal attention and frontal parietal networks in working memory and cognitive control processes [89], these two networks did not stand out as the most important networks in our analyses. An explanation for these findings remains unclear.

### Considerations and future study

A major strength of the current study design was the inclusion of both cognitive science and psychometric tests to form broad ability factors, a potentially more stable set of measures of individual differences than individual tasks [90]. Furthermore, executive function and episodic memory are often seen as two of the most important domains in cognitive aging, with well-known neurobiological underpinnings [49]. Other approaches have elucidated reference cognitive dimensions strongly linked to executive function and episodic memory [58,91], and could be used as external validation for the current study’s predictive models and spatial pattern of results [23,92]

In terms of the predictive modeling approach, there are more complex algorithms available (e.g., support vector regression) and other dimension reduction approaches for dealing with high dimensional data such as regional morphometry data (e.g., lasso or principal component regression). However, univariate feature selection, followed by averaging, and linear regression estimation was specifically used in a theoretically driven extension to previous studies’ univariate whole brain analytical approaches [25,38,39]. Finding an algorithm with the largest predictive ability was not a priority. However, given the predictive ability observed in the current study, the simple and highly interpretable linear regression workflow used here can be a useful baseline model in future studies aiming to achieve the highest possible predictive ability (i.e., smallest prediction error).

The below-chance predictive ability for surface area was also noteworthy. While the predictive ability of cortical thickness stemmed from reliable positive relationships with cognition, surface area contained highly variable training coefficients across folds (i.e., training linear regression weights varied between positive and negative), contributing to below average predictive ability with the current linear regression algorithm. It may be useful in future cortical structural predictive modeling studies to separate weights for positive and negative vertices. However, as stated previously, we did not create more complex steps in our analysis pipeline (e.g., differentiating between positive and negatively related regions) due to our theoretical focus on the combined strength of functional networks. However, as evident from the exploratory whole brain analyses, thickness exhibited many more regional associations regardless of the direction of the relationship, evidence that adding this step would not dramatically effect results.

Finally, functional connectivity of the salience and default mode network have been linked to preclinical biomarkers of structural pathology [93]. We did not screen for such preclinical pathology that could go undetected through MRI screenings (e.g., the MMSE used in the current study) or diagnosed pathologies [94]. Although the mediating influence of global biomarkers of pathology, cortical thickness, and functional connectivity has been investigated [55], a network-based approach to multiple biomarkers of these networks may help specify the mechanisms of aging and age-related disease.

Importantly, these results set the stage for linking cortical morphometry to lifestyle factors and fitness intervention-related changes sensitive to individual differences in patterns of functional integrity [3,5]. Extensive research has shown cortical volume (a product of surface area and thickness but more related to surface area) changes via voxel based morphometric measurements [95,96], but cortical thickness may be more sensitive to experience-related effects such as employment during retirement or fitness [97–99]

### Conclusions

We used functional network maps as an organizing principle to guide an investigation of individual differences in cortical structure, cognition, and age in older adults. Our investigation found morphometric phenotype should be taken into account when age may play a role in individual differences, such as found in older adults. Specifically, thickness, rather than surface area, was most associated with age-related cognition. In terms of individual functional networks, structural integrity of the default mode and somatomotor network may be particularly important for general age-related abilities. The salience network may be important specifically for executive function. The current study has introduced approaches to using functional networks to guide cortical structural analyses rather than simply using functional network maps as tools for *post hoc* interpretations. Future work should include related cognitive abilities with other samples in order to extend conclusions derived from the current analyses.

## Supporting information

S1 File Document containing supplemental figures and tables. Figure A. Distribution of the total number of vertices selected across all 500 fold training sets (5 folds, 100 iterations) and collapsed across all alpha thresholds (.05,.01,.001,.0005,.0001) for each model type. Note, vertices were selected based on their univariate association with cognition. The networks are color coded and ordered according to the parcellation scheme used(visual = 1 (violet), somatomotor = 2 (blue), dorsal attention = 3 (green), salience = 4 (fuchsia), limbic = 5 (cream), control = 6 (orange), default mode = 7 (red))
Figure B. The percentage of folds (5 folds, 100 iterations) with no vertices selected for each network and alpha threshold (0.05, 0.01, 0.001, 0.0005, 0.0001) . The networks are color coded and ordered according to the parcellation scheme used (visual = 1 (violet), somatomotor = 2 (blue), dorsal attention = 3 (green), salience = 4 (fuchsia), limbic = 5 (cream), control = 6 (orange), default mode = 7 (red))
Figure C. Unthresholded statistical maps of the bootstrap ratio scores for the relationship of cognition and morphometry.
Figure D. Unthresholded statistical maps of the bootstrap ratio scores for the mediation effect of the age and cognition relationship.
Figure E. Thresholded statistical maps of the bootstrap ratio scores for the relationship of age and morphometry.
Figure F. The observed bivariate correlation (r) values (i.e., the mean correlation coefficient across bootstrapped samples) for all pairs involved in the previous whole brain analyses as well as cortical volume (i.e., product of surface area and thickness).
Figure G. Pearson correlation coefficients (mean across 2000 bootstrap replicates) showing the relationships between cortical structure metrics used in the current manuscript as well as both these phenotypes’ relationships with volume.
Table A. Correlations (r) age, cognition, and morphometry across entire cortex.
(DOCX)

S2 File Dataset containing prediction model results. (CSV)

S3 File Dataset of average predictions for each subject and network from predictive models. (CSV)

S4 File Dataset containing the composite scores, individual task scores, and summary structural measures across the entire cortex. (XLSX)
