# Supplementary material for: The cortical structure of functional networks associated with age-related cognitive abilities in older adults
Source: PLoS One. 2018 Sep 21;13(9):e0204280. doi: 10.1371/journal.pone.0204280 (PMC6150534; doi:10.1371/journal.pone.0204280)
Supplement: S1 File — Figure A. Distribution of the total number of vertices selected across all 500 fold training sets (5 folds, 100 iterations) and collapsed across all alpha thresholds (.05,.01,.001,.0005,.0001) for each model type. Note, vertices were selected based on their univariate association with cognition. The networks are color coded and ordered according to the parcellation scheme used(visual = 1 (violet), somatomotor = 2 (blue), dorsal attention = 3 (green), salience = 4 (fuchsia), limbic = 5 (cream), control = 6 (orange), default mode = 7 (red)) Figure B. The percentage of folds (5 folds, 100 iterations) with no vertices selected for each network and alpha threshold (0.05, 0.01, 0.001, 0.0005, 0.0001) . The networks are color coded and ordered according to the parcellation scheme used (visual = 1 (violet), somatomotor = 2 (blue), dorsal attention = 3 (green), salience = 4 (fuchsia), limbic = 5 (cream), control = 6 (orange), default mode = 7 (red)) Figure C. Unthresholded statistical maps of the bootstrap ratio scores for the relationship of cognition and morphometry. Figure D. Unthresholded statistical maps of the bootstrap ratio scores for the mediation effect of the age and cognition relationship. Figure E. Thresholded statistical maps of the bootstrap ratio scores for the relationship of age and morphometry. Figure F. The observed bivariate correlation (r) values (i.e., the mean correlation coefficient across bootstrapped samples) for all pairs involved in the previous whole brain analyses as well as cortical volume (i.e., product of surface area and thickness). Figure G. Pearson correlation coefficients (mean across 2000 bootstrap replicates) showing the relationships between cortical structure metrics used in the current manuscript as well as both these phenotypes’ relationships with volume. Table A. Correlations (r) age, cognition, and morphometry across entire cortex. (DOCX) [file pone.0204280.s001.docx]

# Supplemental Figures


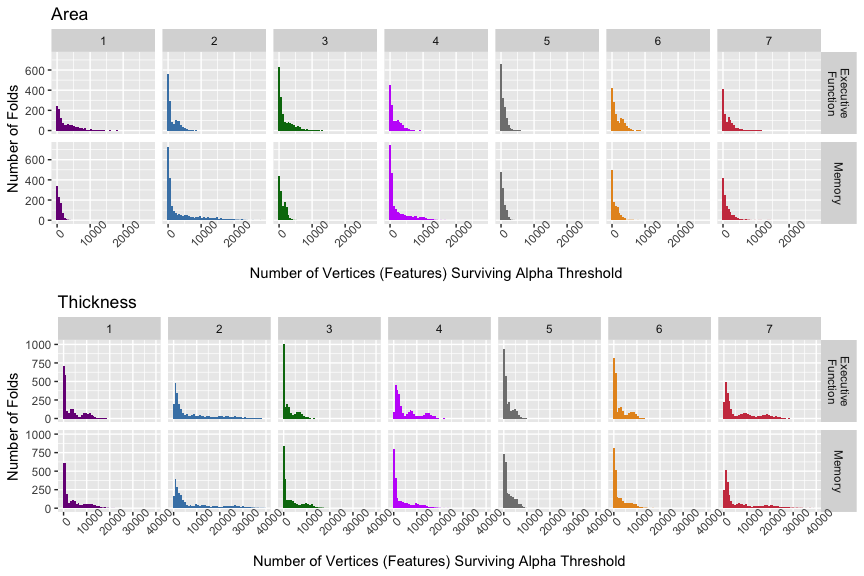


Figure A. Distribution of the total number of vertices selected across all 500 fold training sets (5 folds, 100 iterations) and collapsed across all alpha thresholds (.05,.01,.001,.0005,.0001) for each model type. Note, vertices were selected based on their univariate association with cognition. The networks are color coded and ordered according to the parcellation scheme used(visual=1 (violet), somatomotor=2 (blue), dorsal attention=3 (green), salience=4 (fuchsia), limbic=5 (cream), control=6 (orange), default mode=7 (red))


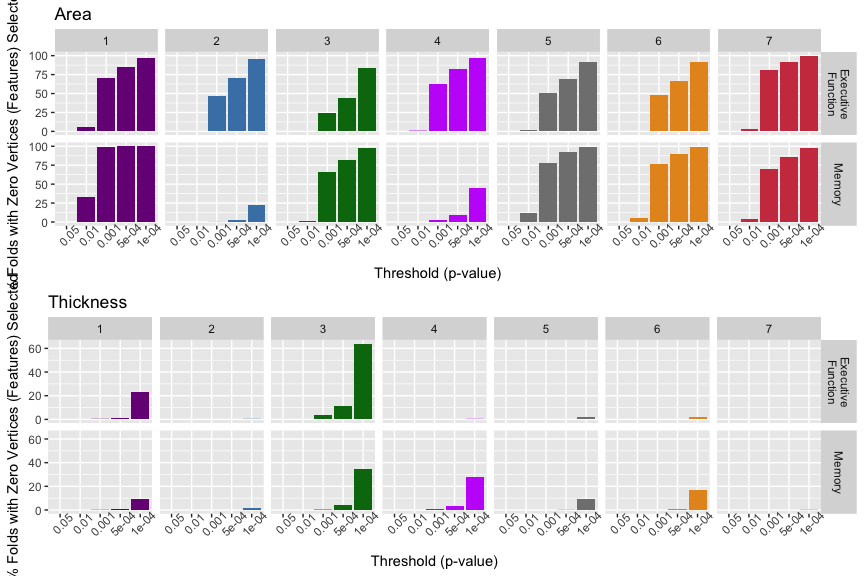


Figure B. The percentage of folds (5 folds, 100 iterations) with no vertices selected for each network and alpha threshold (0.05, 0.01, 0.001, 0.0005, 0.0001) . The networks are color coded and ordered according to the parcellation scheme used (visual=1 (violet), somatomotor=2 (blue), dorsal attention=3 (green), salience=4 (fuchsia), limbic=5 (cream), control=6 (orange), default mode=7 (red))


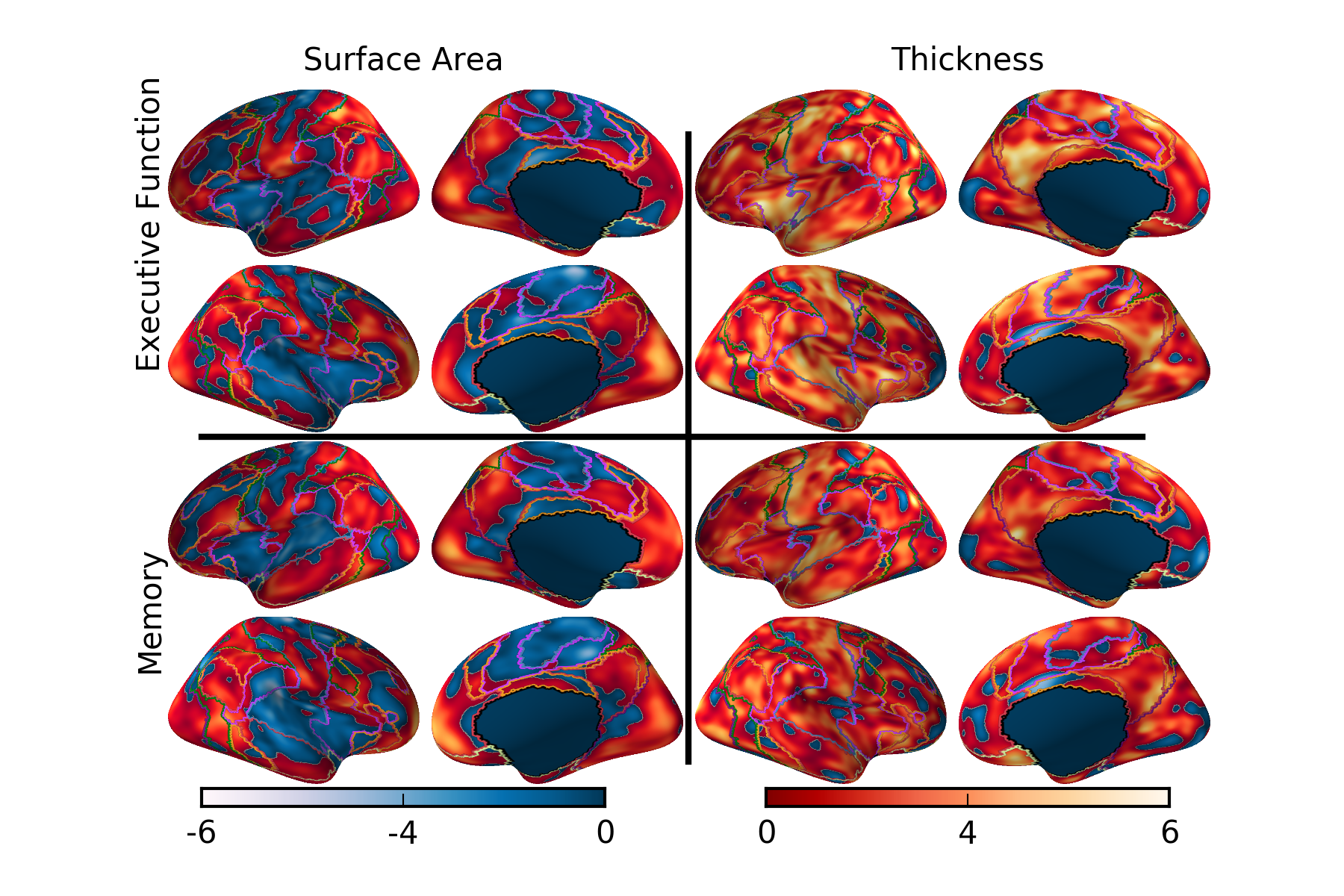


Figure C. Unthresholded statistical maps of the bootstrap ratio scores for the relationship of cognition and morphometry.


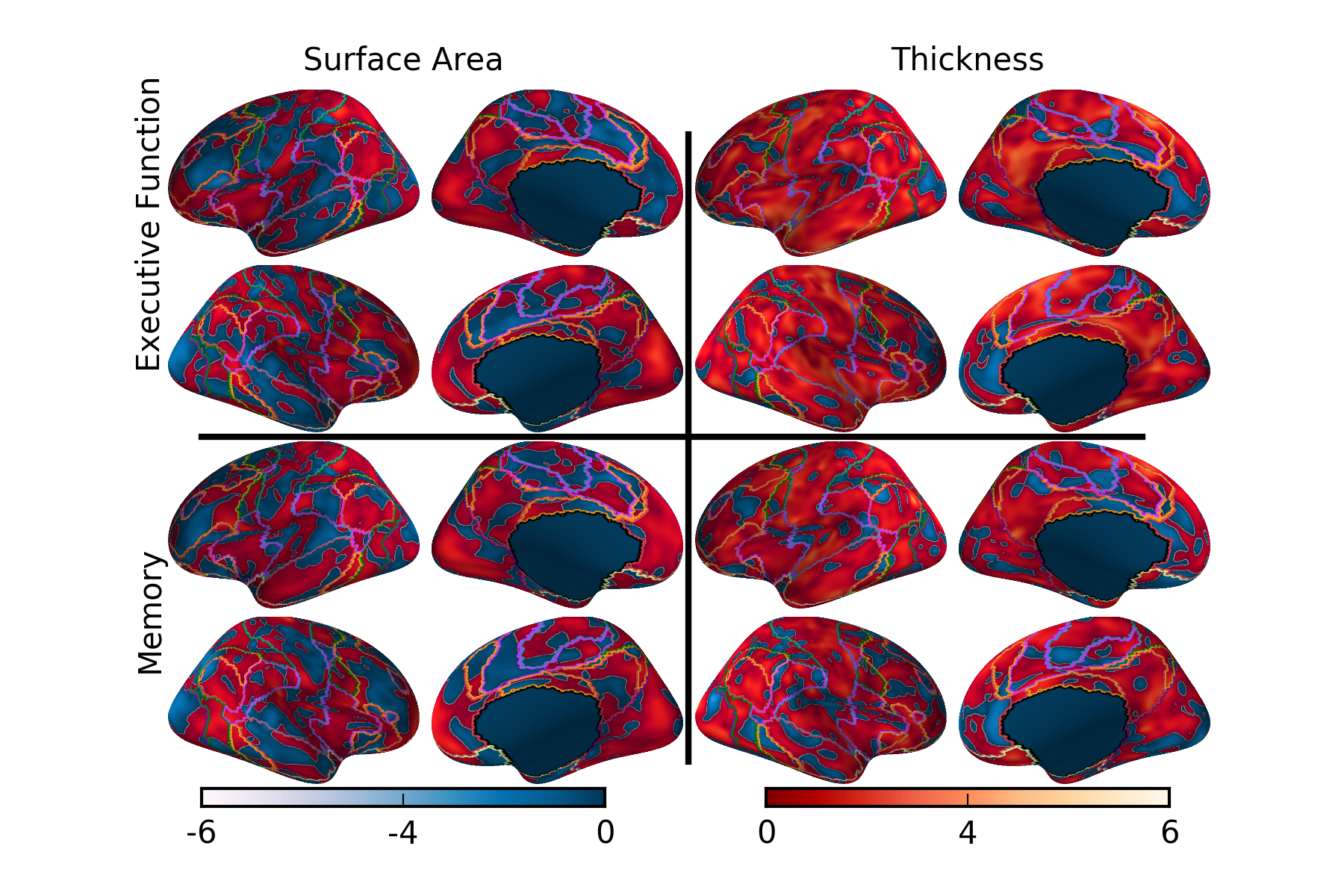


Figure D. Unthresholded statistical maps of the bootstrap ratio scores for the mediation effect of the age and cognition relationship.

*
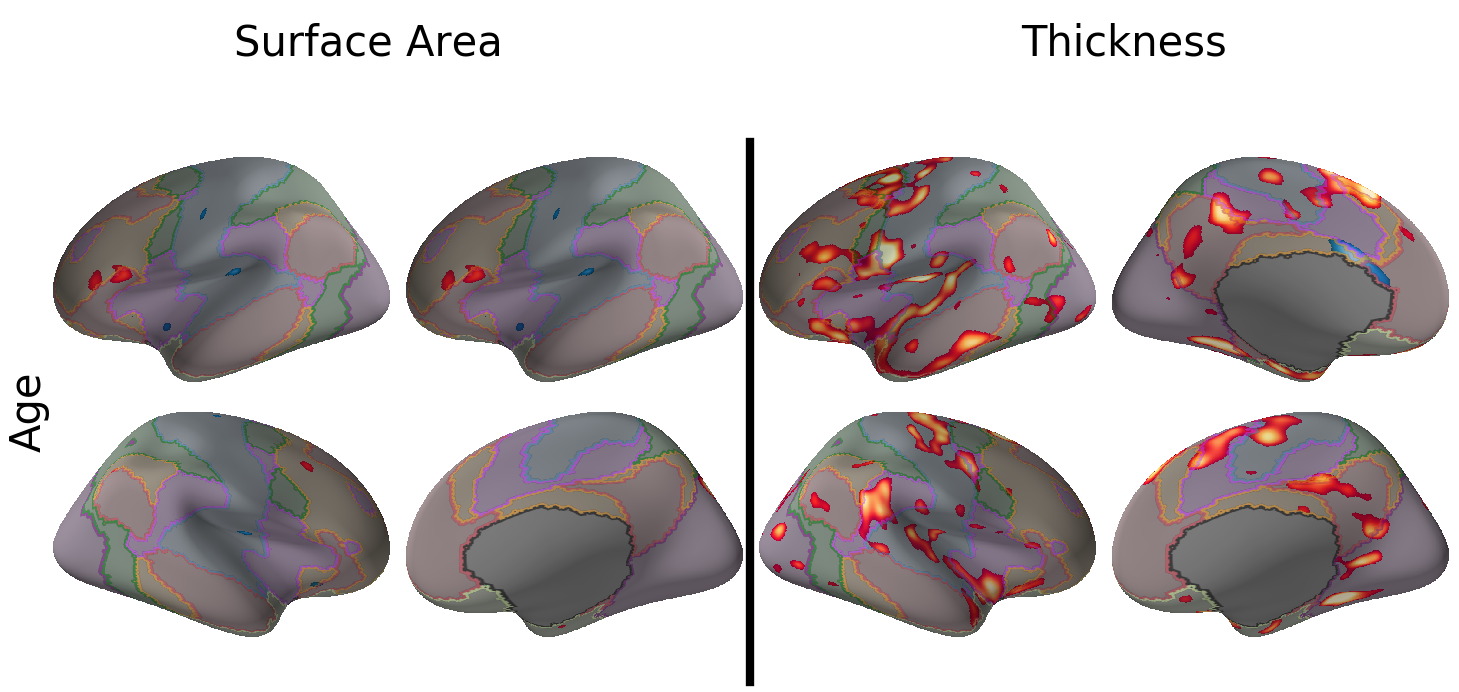
*

Figure E. Thresholded statistical maps of the bootstrap ratio scores for the relationship of age and morphometry.

Table A. Correlations (r) age, cognition, and morphometry across entire cortex

|  | Age | Executive Function | Memory | Average Thickness | Total Surface Area | Total Volume |
| --- | --- | --- | --- | --- | --- | --- |
| Age | 1.00 | -0.30 | -0.37 | -0.35 | -0.02 | -0.20 |
| Executive Function | -0.30 | 1.00 | 0.43 | 0.28 | 0.07 | 0.23 |
| Memory | -0.37 | 0.43 | 1.00 | 0.29 | -0.09 | 0.09 |
| Average Thickness | -0.35 | 0.28 | 0.29 | 1.00 | -0.31 | 0.26 |
| Total Surface Area | -0.02 | 0.07 | -0.09 | -0.31 | 1.00 | 0.83 |
| Total Volume | -0.20 | 0.23 | 0.09 | 0.26 | 0.83 | 1.00 |

*Note. All morphometry variables refer to the average or total across the entire cortex.*

*
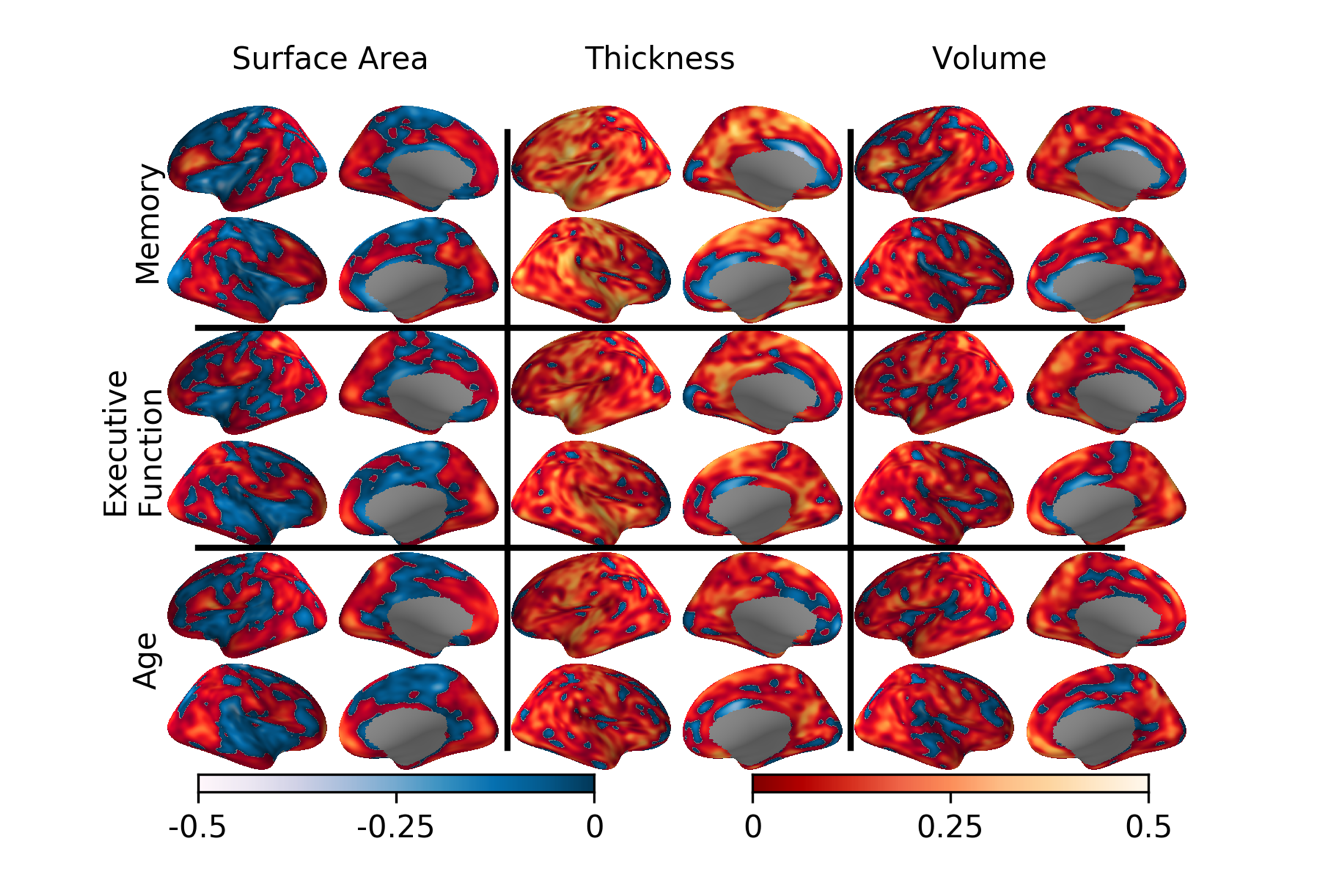
*

Figure F. The observed bivariate correlation (r) values (i.e., the mean correlation coefficient across bootstrapped samples) for all pairs involved in the previous whole brain analyses as well as cortical volume (i.e., product of surface area and thickness).

*
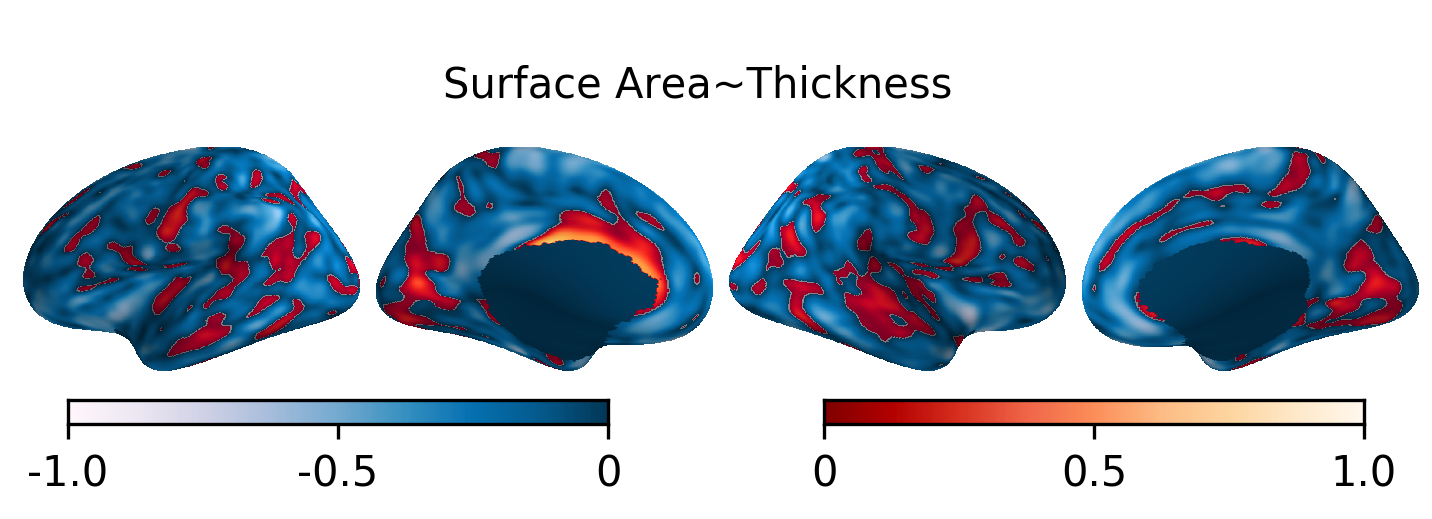
*

*
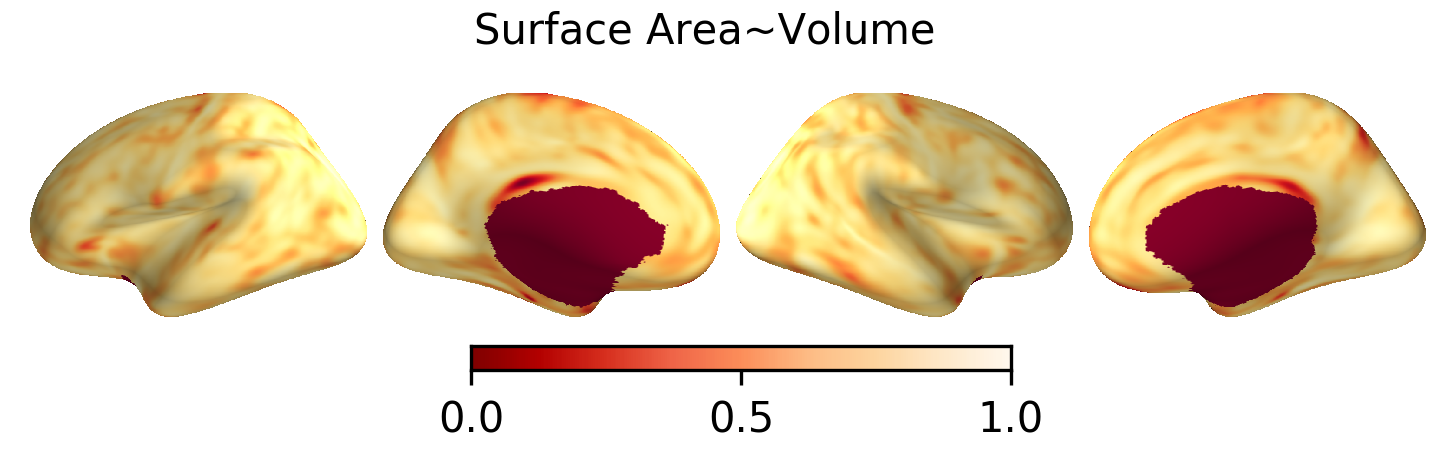
*

*
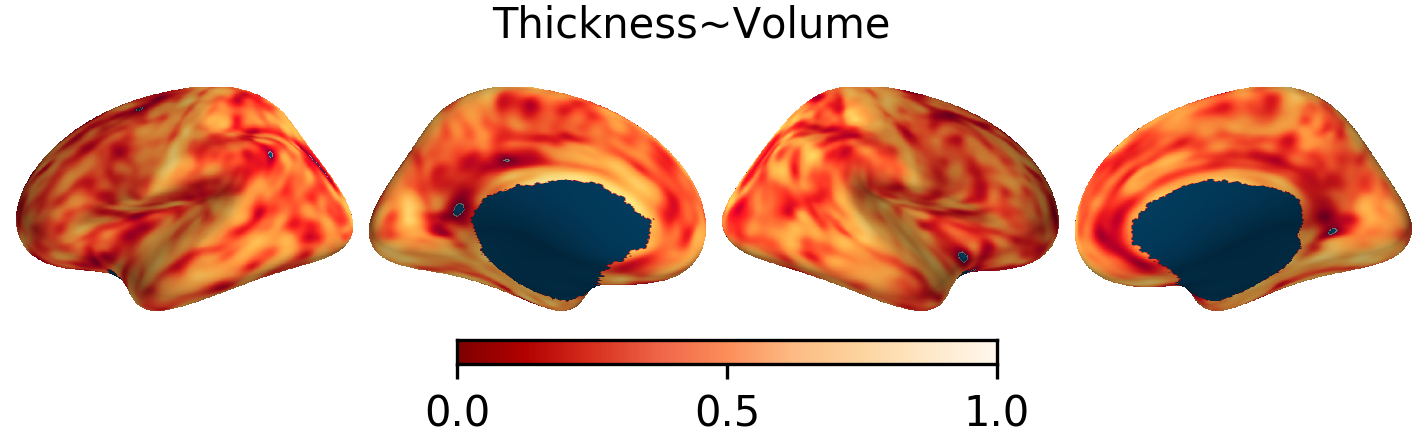
*

Figure G. Pearson correlation coefficients (mean across 2000 bootstrap replicates) showing the relationships between cortical structure metrics used in the current manuscript as well as both these phenotypes’ relationships with volume.
